# Mr. Cooper, in Reply to Mr. Dawson

**Published:** 1805-09-01

**Authors:** W. O. Cribb

**Affiliations:** Bishops Stortford, Herts


### *To the Editors of the Medical and Physical Journal.*

Gentlemen,

I Have to acknowledge the very liberal and handsome manner in which Mr. Dawson has offered some "*conjectural remarks*" on the singular case of delivery, inserted in page 17 of your present volume; and you will permit me to observe, that if every controversy, of which your Journal has been made the vehicle, had been conducted with the same modesty and candour, some of your readers would not so often have been offended with the rancour and asperity which have distinguished, as I think, too many of the papers in your valuable miscellany.

It is often difficult, and frequently impossible, to describe on paper every *feeling* and *appearance* we notice, in cases which it may be proper to lay before the profession, either as curious for the physiologist or useful to the practitioner, so as to make them clear to every perception. To this source I must attribute the misconception of Mr. Dawson, and the erroneous conclusions he has deduced therefrom. His argument is founded in error, from not considering the exact point from which the examination was first made; he does not seem aware that when I described the tumour, "as covered by an unusual substance, which felt *soft* and *uneven* to the touch," that my my finger was then, *unknowingly*, in the *rectum*; and had he proceeded a little further on in the page, he would have found an insurmountable difficulty to his *conjecture*; viz. "that the projection of the *os sacrum* was much more plainly perceived than in a common examination;" and still lower in the same page, he would have seen all the obscurities of this first examination, "*satisfactorily accounted for*;" and *why* the os uteri and arch of the pubis were not to be felt. If this conjecture of Mr. Dawson's could be for a moment allowed, it would not, in my opinion, afford any of the criteria of this examination; the tumour was hard, very hard, allowing me to press on it firmly, so as to be satisfied it was the head of a child; and I could, through the *soft* and *uneven* covering, indistinctly feel the yielding of the bones. Had this been "*an unnatural thickening and resisting state of the membranes investing the fœtus*," the perception to the finger must have been very different; this "unnatural thickening and resisting state," would have prevented my so readily feeling the head, and the membranes would then have felt *firn* and *smooth*, instead of *soft* and *uneven*. Again I urge, if it was this unnatural membrane I was pressing against, it must either have been high up, or low down; if the former, there would have been no difficulty in finding a pervious vagina; and if so low down as "completely to wedge up the vagina," how could the projection of the sacrum be so easily perceived, as to make it a prominent difficulty in ascertaining the nature of the case?

Now all this *conjectural cloud* vanishes by taking the case as it really stands described and explained in my last. The soft and uneven substance is there stated to be the *intestinum rectum et vagina*, and the difficulty in not being able to discover the *os uteri* and arch of the pubis is clearly accounted for. Mr. D. also *conjectures*, that if there had been an adhesion of the sides of the vagina, "there would, at least, have been half an inch in length of the sides of the *vagina* united;* this I am not disposed to controvert, but who will say, what alteration might have taken plase in this thickening, between Tuesday and Friday, during the whole of which period the

* It is more than probable that the projection mentioned, p. 18, as immediately under the meatus urinarius, might be this thickening and adhesion, and which is described as a "pushing out of a portion of the *vagina*."

woman was in strong labour, and for a great part of the time the head of the child was within the brim of the pelvis, and of course, the vagina greatly on the stretch? But there is still an insurmountable difficulty to Mr. Dawson's *conjectural hypothesis.* He does not take into consideration, that Mr. Cooper was with this woman before I saw her; indeed, he was there early in the beginning of labour, and it is stated (page 18) "that the same circumstance" (passing the finger into the rectum, and not being able to find the vagina) "had constantly happened to him during the whole time he had attended her." Now, how comes it to pass, that this "surprisingly thickened, unnatural, and resisting state of the membranes," should obliterate the vaginal aperture in the beginning of labour, and before they could so "*completely wedge it up as that the hand could not pass?*" Alas! Gentlemen, how may a little fanciful conjecturing bewilder the imagination!

Mr. Dawson also *conjectures,* that the woman seemed to have no suspicion of the circumstances attending her case. She certainly was all along aware of it, and it was to this alone that the mother alluded, when she replied to Mr. Cooper's enquiries, that her "*daughter was just as she was,*" in point of delivery.

Having thus noticed the principal points of Mr. Dawson's conjectural observations, permit me to encroach a few moments on your patience, while I retrace, as shortly as possible, the leading features of the case, and which will tend to show, that we could not be so egregiously mistaken, as we have been supposed by Mr. D.

When Mr. Cooper visited her, under a suppression of urine, and passed the catheter, he "*found*" the vagina impervious; Mr. Dawson has it, "he *fancied* the vagina to be impervious." Here, indeed, Mr. D's candour and fairness seem a little to have forsaken him. When again Mr. C. was called to attend her in labour, he went with his attention roused, as to how this curious business would terminate; and he then found, as he had heretofore repeatedly done, the vagina impervious, or rather obliterated as to any external opening. He, however, waited a considerable time, to see if the parts would so far dilate, by the natural efforts, as that he might find an aperture; and seeing no probability of her being relieved without assistance, he requested Mr. Tice's and my attendance; and had the precaution to let us form *our own* opinions of the case, from repeated examinations, (that we might be unbiassed) before he related to us what he had before known and and observed. Had we even rested here, and trusted to the *touch*, it might have been said we were deceived; but that nothing might be left undone to acquaint ourselves with the exact nature of the case, the parts were submitted to *inspection*, under the most favourable circumstances, and yet no opening found. To suppose that three professional men, thus situated, who were extremely cautious and circumspect, could *possibly* mistake "an unnatural thickening and resisting state of the membranes" for an imperforation* of the vagina, appears to me a very uncandid conjecture; and I must be free to say, had that been really the case, neither of them were fit to practice; for I cannot accept Mr. Dawson's apology, p. 155, that it was, " a misapprehension, which, under similar circumstances, many men might have fallen into," as, in my opinion, no person, possessing proper intelligence for practice, could possibly have made so palpable a blunder.

From this short recapitulation, and a re-perusal of the case in question, I think it will appear, that it was, as I have related it, an *imperforated vagina;* arising most probably from ulceration and adhesion after the former labour. Had the case indeed occurred to me in my private practice, and not witnessed by an other practitioner, I certainly should not have ventured to brave the scepticism of my brethren by a description of it; but, supported as I am, by the opinion and presence of two such respectable and intelligent coadjutors, I feel confident, whatever my individual deficiencies may be, that these Gentlemen were not likely to be easily deceived, and that too by such an appearance as Mr. Dawson conjectures and describes. Whatever deception we may have laboured under, it was assuredly not of the nature which Mr. D. has pointed out, as, independent of my relation of the case not being at all adapted to, or to be explained by, this conjecture of his, I have no hesitation in affirming, we could not thus have been deceived; for, in that case, as in all others of *protrusion*, whether of the membranes, the occiput, or the nates, do we not instantly perceive, " notwithstanding the vagina may be completely wedged up," the *margin* of the stretched os externum? yet, in this case, the external parts were scarcely altered, except by that small projec-

* I use the word imperforation, (though not strictly proper) as being [illegible] and expressive.

tion mentioned in page 18, and alluded to in the first note of this paper.

Having trespassed so long on your time and attention, I shall refer Mr. Dawson to the conclusion of my former letter, respecting his observations on the escape of the *liquor amnii*, and the menstrual discharge; they are such as must strike every intelligent physiologist; they were what occurred forcibly to our minds at the first; and in drawing up the case, I carefully avoided "*wandering into the regions of* CONJECTURE, *or building a principal hypothesis on so uncertain a foundation.*" I shall pursue the same course at present, with this single observation, that it was *our conjecture* at the time, that there was, although we had not discovered it, some connection between the rectum and vagina; but as we had no opportunity of examination post mortem, I purposely avoided all *conjectural reasoning*, and presented such facts and appearances only, as were really observed, and for the accuracy and fidelity of which we could readily vouch.

With again acknowledging Mr. Dawson's candour, and assuring him that no offence can be taken at the manner in which he has brought it forward, I shall take a *final* leave of the Controversy, with requesting the Editor to correct a verbal inaccuracy in page 18, l. 15, where, for "a year before," read nine years before.

I am, &c.

W. O. CRIBB.

*Bishops Stortford, Herts, Aug.* 3, 1805.

---

*The following Letter from Mr. Cooper, will throw some additional light on this curious Case.*

"Dear Sir,

"I have carefully perused your answer to Mr. Dawson's *conjectures*, in the last Number of the Medical and Physical Journal; and have only to add to your very satisfactory Reply, that I had more than once an opportunity of examining the vagina, when the woman was not in a state of pregnancy, and could never pass my finger, although I repeatedly attempted it, and which could not, then, have been caused by any 'thickening or resisting state of the membranes;' and this I did by desire of the mother, who always when I saw her, or at least if her daughter ailed any thing, was very solicitous about her on account of this, her unnatural situation, as she always thought it. Neither was the woman herself, or the husband, band, ignorant of it; and if it was consistent with *delicacy* and *propriety*, to publish what *is known* on this head, it would place the case beyond the possibility of doubt. When I first saw her in labour, I could find no aperture to the vagina; and the *readiness* with which the finger passed into the rectum, is not to be conceived but by those who had an opportunity of examining; at this period the head of the child was not to be felt, it had not passed within the brim of the pelvis; when it did, the examination *per anum* soon discovered it.

"I am sorry that Mr. Dawson's exuberant imagination should give you so much trouble, as I think your former paper on this subject sufficiently clear and explicit.

"I am, dear Sir,

"Your's, truly,

*Much Hadham, Herts, Aug.* 5, 1805. "G. Cooper."

"To Mr. Cribb."

