# Erratum

**Published:** 1805-09-01

**Authors:** 


### ERRATUM.

P. 242—245, Running Title, for Mr. Cooper, read Mr. Cribb.

