# Errata

**Published:** 1805-10-01

**Authors:** 


### ERRATA.

P. 244, l. 23, *just as she was, in point of delivery,* dele *in point of delivery.*

P. 246, l. 10, for *principal* read *fanciful.*

